# Conceitos atuais no tratamento das fraturas distais do fêmur no adulto

**DOI:** 10.1055/s-0046-1822640

**Published:** 2026-06-08

**Authors:** Fabricio Fogagnolo, Ricardo Antônio Tavares

**Affiliations:** 1 Departamento de Ortopedia e Anestesiologia, Hospital das Clínicas, Faculdade de Medicina de Ribeirão Preto, Universidade de São Paulo, Ribeirão Preto, SP, Brasil

**Keywords:** fraturas de Hoffa, fraturas do fêmur, joelho, osteoporose, femoral fractures, Hoffa fracture, knee, osteoporosis

## Abstract

As fraturas distais do fêmur representam aproximadamente 1% de todas as fraturas e 3 a 7% das fraturas femorais, caracterizando-se como lesões complexas que frequentemente envolvem a superfície articular. A tomografia computadorizada é fundamental na avaliação da extensão articular, de eventuais traços coronais e da cominuição metafisária. O tratamento cirúrgico busca restaurar o alinhamento mecânico, a congruência articular e promover estabilidade suficiente para mobilização precoce.

A epidemiologia apresenta padrão bimodal: em jovens, predominam traumas de alta energia; em idosos, prevalecem quedas de baixa energia, com maior mortalidade devido à osteoporose e às comorbidades. O tratamento conservador possui indicações restritas, sendo normalmente reservado a pacientes com alto risco cirúrgico ou fraturas não desviadas.

O tratamento cirúrgico inclui placas bloqueadas, hastes intramedulares retrógradas e técnicas combinadas. A dupla fixação tem ganhado destaque em ossos osteoporóticos, perda de suporte medial e fraturas periprotéticas. Em casos selecionados, especialmente em idosos com osso muito fragilizado, a artroplastia do joelho pode ser uma alternativa para possibilitar mobilização precoce. Este artigo apresenta uma revisão atualizada dos principais aspectos epidemiológicos, diagnósticos e terapêuticos das fraturas distais do fêmur, incluindo situações especiais, como as fraturas expostas, fraturas de Hoffa e as fraturas periprotéticas.

Complicações como infecção, pseudoartrose, rigidez e osteoartrite pós-traumática ainda são frequentes.

## Abstract (en)

Distal femur fractures account for approximately 1% of all fractures and 3 to 7% of femoral fractures, representing complex injuries that frequently affect articular surfaces. Computed tomography is essential to evaluate articular involvement, the presence of coronal fracture lines, and metaphyseal comminution. Surgical treatment aims to restore mechanical alignment and articular congruity while providing sufficient stability to allow early mobilization.

The epidemiology shows a bimodal pattern: in younger patients, high-energy trauma predominates, whereas in older patients, low-energy falls are more common, with higher mortality related to osteoporosis and comorbidities. Conservative treatment has limited indications and is often reserved for patients with high surgical risk or for nondisplaced fractures.

Surgical management includes locking plates, retrograde intramedullary nails, and combined fixation techniques. Dual fixation has gained increasing attention in osteoporotic bone, cases with loss of medial support, and periprosthetic fractures. In selected cases, particularly in older patients with severely compromised bone quality, knee arthroplasty may be an alternative for early mobilization. The present article presents an updated review of the main epidemiological, diagnostic, and therapeutic aspects of distal femur fractures, including special situations such as open fractures, Hoffa fractures, and periprosthetic fractures.

Complications such as infection, nonunion, stiffness, and posttraumatic osteoarthritis remain frequent.

## Introdução

As fraturas distais do fêmur correspondem a cerca de 1% de todas as fraturas e a aproximadamente 3 a 7% das fraturas femorais. 1 2 A tomografia computadorizada (TC) desempenha papel fundamental, pois ela identifica traços coronais posteriores, determina o grau de cominuição metafisária e orienta a escolha da estratégia operatória. O planejamento cirúrgico, por sua vez, deve considerar a restauração do alinhamento mecânico tanto no plano frontal como no sagital, a reconstrução anatômica da superfície articular e a obtenção de estabilidade suficiente para permitir mobilização precoce. O advento das placas bloqueadas, das hastes intramedulares retrógradas e das fixações combinadas 3 4 5 6 ampliou de forma significativa o arsenal terapêutico, permitindo melhor fixação em padrões complexos de fraturas, mesmo com qualidade óssea ruim e com possibilidade de descarga de peso adequada às condições clínicas do paciente. 7 8 9 Apesar dos avanços, persistem taxas relevantes de complicações, como rigidez, pseudoartrose, falha do implante, infecção e osteoartrite pós–traumática.

## Epidemiologia e mecanismos de trauma

As fraturas distais do fêmur apresentam clara distribuição epidemiológica bimodal. 2 Em adultos jovens, predominam os traumas de alta energia. Nesses pacientes, é comum a presença de fraturas expostas, lesões ligamentares complexas, luxações traumáticas do joelho, comprometimento neurovascular e lesões associadas, o que aumenta significativamente o risco de complicações. 10 A magnitude da energia envolvida também contribui para maior incidência de fraturas articulares completas, incluindo traços coronais posteriores (fraturas de Hoffa). 11 Em idosos, o mecanismo predominante é o trauma de baixa energia, geralmente decorrente de queda da própria altura (perfazendo 61% dos casos). 1 Neste grupo, encontramos uma maior prevalência de fraturas entre as mulheres. A presença de osteoporose, sarcopenia e múltiplas comorbidades contribui para padrões de fratura com cominuição metafisária, fragilidade da cortical óssea e maior risco de falha de fixação. A mortalidade nessa população é elevada, podendo alcançar de 26 a 30% no 1° ano após a fratura 3 12 13 14 15 , índice comparável ao observado em fraturas do quadril. Nos traumas de baixa energia em idosos, o desafio central é operar precocemente pacientes em idade avançada com comorbidades, permitindo mobilização articular sem risco de falha do implante. Esse panorama reforça a necessidade de abordagem individualizada, para a definição da estratégia terapêutica ideal.

## Diagnóstico

O diagnóstico das fraturas distais do fêmur requer abordagem sistemática. A inspeção pode revelar deformidade, encurtamento, rotação externa do membro, edema e, em casos de alta energia, fraturas expostas ou sinais indiretos de lesões articulares. A avaliação neurovascular é obrigatória, dada a proximidade das estruturas poplíteas. Lesões arteriais podem se apresentar como pulso ausente, enchimento capilar reduzido, extremidade fria ou como simples assimetria nos pulsos tibiais posteriores e pediosos. Em casos suspeitos, Doppler, angiotomografia ou consulta imediata à cirurgia vascular são essenciais. Quando expostas, tais fraturas guardam prognóstico mais reservado, devido ao risco aumentado de infecção, maior número de cirurgias secundárias, índices maiores de retardes de consolidação e de pseudoartroses. Podemos encontrar também lesões parciais ou totais do tendão do quadríceps, geralmente causadas por uma lesão direta a partir do fragmento proximal desviado anteriormente.

Radiografias iniciais em incidências anteroposterior (AP) e de perfil são indispensáveis e permitem identificar o padrão geral da fratura, mas a avaliação completa da fratura depende da TC para avaliar a extensão intra-articular (em torno de 55% destas fraturas são intra-articulares 16 ), eventuais traços ocultos e a correta identificação das fraturas de Hoffa.

O diagnóstico completo deve integrar todos esses elementos, garantindo que o cirurgião compreenda o padrão ósseo da fratura, o estado das partes moles e a presença de lesões associadas que possam interferir na estratégia terapêutica.

## Classificação

O sistema Arbeitsgemeinschaft für Osteosynthesefragen/Orthopedic Trauma Association (AO/OTA) é amplamente adotado para a classificação das fraturas distais do fêmur, sendo que as fraturas do tipo A são as extra-articulares, as do tipo B são articulares parciais e as do tipo C são as fraturas articulares totais, quando não há nenhuma contiguidade entre a diáfise e a superfície articular. As fraturas da região distal do fêmur podem ter impacto significativo sobre a articulação do joelho, pois desvios relativamente pequenos no plano frontal ou sagital têm impacto substancial no eixo mecânico do membro, podendo resultar em osteoartrite pós-traumática no longo prazo.

## Indicações do tratamento conservador

O tratamento conservador é atualmente reservado a um grupo muito restrito de pacientes, e a literatura é pobre na comparação entre tratamento cirúrgico e conservador. 17 Com a evolução das técnicas de osteossíntese, o manejo não operatório tornou-se exceção, e trabalhos recentes mostram menor mortalidade com o tratamento cirúrgico em pacientes idosos. 18

Excetuando-se a população pediátrica, na qual o tratamento conservador com gesso ou tração é indicado em algumas situações, são raros os casos em que o tratamento cirúrgico não é indicado em pacientes adultos, situação em que podemos enquadrar pacientes mais idosos que não deambulam e aqueles que possuem alto risco cirúrgico por comorbidades, pacientes com infecção ativa no membro acometido ou casos de fraturas não desviadas. Mesmo nestes casos, a fixação externa poderia ser considerada. É extremamente difícil imobilizar fraturas femorais com talas, órteses ou tutores externos e o tratamento cirúrgico tem a função de promover conforto e reduzir complicações clínicas e locais, mesmo em pacientes limitados funcionalmente.

## Tratamento cirúrgico

### Momento da cirurgia

A definição do momento ideal para a cirurgia exige avaliação criteriosa da condição clínica do paciente, do estado das partes moles e da presença de lesões associadas. Para pacientes com fraturas isoladas, o tratamento definitivo pode ser planejado e realizado já nos primeiros dias. Em situações de trauma de alta energia, é comum que o paciente apresente lesões de partes moles ou edema acentuado, sendo indicado o uso de fixadores externos para estabilização temporária. Em politraumatizados, a osteossíntese definitiva é realizada quando o paciente atinge a chamada “janela da oportunidade”, 5 a 10 dias após o trauma, momento em que parâmetros hemodinâmicos e metabólicos estão estabilizados. Em pacientes idosos com fraturas de baixa energia, o atraso cirúrgico prolongado associa-se à maior taxa de complicações, e atrasos superiores a 2 ou 3 dias aumentam significativamente a morbimortalidade. 3 14 Por serem fraturas complexas, demandam estudo prévio das imagens e das condições clínicas para o planejamento cirúrgico, incluindo não apenas a definição do momento da cirurgia, mas também a escolha das vias de acesso, táticas de redução, escolha dos implantes e métodos de fixação.

## Planejamento cirúrgico

O posicionamento do paciente varia conforme a via de acesso e o padrão da fratura. Embora a posição supina seja a mais comum, fraturas de Hoffa posteriores podem demandar decúbitos alternativos ou abordagens combinadas. Eventualmente, o decúbito pode necessitar ser modificado durante a cirurgia ( *floating position* ). A exposição deve permitir visualização e redução direta dos traços articulares e redução indireta da metáfise. Distratores e clampes periarticulares facilitam a redução e devem estar à disposição. A superfície articular deve ser reduzida anatomicamente e estabilizada com compressão interfragmentária; já a metáfise exige estabilidade relativa. Parafusos distais devem respeitar a linha de Blumensaat (região intercondilar) e incidências em 25° de rotação interna permitem avaliação do tamanho dos parafusos no côndilo medial. A necessidade de enxertia óssea deve ser antecipada. Sua utilização é historicamente recomendada nos casos de falhas ósseas importantes ou ausência de contato medial. Recentemente, com o advento da fixação com um segundo implante medial, não existe uma recomendação formal para enxertia óssea. Um segundo implante medial tem sido indicado em ossos osteoporóticos, fraturas periprotéticas ou em falhas de contato ósseo medial. 7 O alinhamento frontal e sagital deve ser checado repetidamente com intensificador, 19 tomando como referência os valores normais dos ângulos metafisários e o membro contralateral. Como são procedimentos longos, o torniquete deve ser evitado, mas pode ser posicionado proximalmente e insuflado apenas se necessário. Ele não pode dificultar a colocação dos parafusos proximais.

## Vias de acesso e técnicas de redução e fixação

### Fraturas extra-articulares (AO 33A)

As fraturas extra-articulares (AO 33A) podem ser tratadas tanto com placas quanto com hastes intramedulares, que são especialmente vantajosas pela redução indireta e pela inserção percutânea. Como não há comprometimento articular, o objetivo principal é restaurar alinhamento frontal, sagital, axial e o comprimento. A ação dos gastrocnêmios pode exigir antagonização com coxim sob o joelho ou, alternativamente, com pino de Steinmann inserido de anterior para posterior no fragmento distal e tracionado distalmente, assim tensionando a cápsula posterior e prevenindo deformidade em recurvato; esta manobra, descrita por Paccola, 20 deve ser realizada sem o coxim do joelho para evitar antecurvato. A fixação com placas exige rigoroso respeito aos parâmetros anatômicos para evitar invasão da região intercondilar, femoropatelar ou medialização indevida do bloco condilar. Implantes como o dynamic condylar screw (DCS) (Synthes) ou placas bloqueadas devem estar alinhados à diáfise no perfil. Os parafusos distais devem ficar anteriores à linha de Blumensaat. Para minimizar atrito com o trato iliotibial, o implante deve ficar com 10° de rotação interna, e bem adaptado à parede lateral do côndilo. Placas-lâminas anguladas, como as 95°, hoje têm sido utilizadas apenas em osteotomias e foram substituídas pelas placas anatômicas bloqueadas no cenário do trauma agudo.

Na fixação intramedular retrógrada, uma pequena incisão anterior longitudinal permite o acesso ao ponto de entrada, que deve alinhar-se ao canal medular e situar-se cerca de 1 cm anterior ao teto do intercôndilo. A manutenção da redução pode requerer múltiplos parafusos distais de bloqueio e a estratégica colocação de parafusos tipo *poller* . A extremidade distal da haste não deve ficar protrusa, para evitar conflito femoropatelar. 9

### Fraturas articulares unicondilares (AO 33 B1 E B2)

Nas fraturas articulares unicondilares, é necessária uma via de acesso que permita visualização direta do traço articular. Esse padrão frequentemente apresenta cisalhamento sagital com fragmento em cunha e vértice metafisário bem definido. Nesses casos, além dos parafusos de tração perpendiculares ao traço, busca-se estabilidade absoluta com colocação de placa de suporte no vértice, evitando perda secundária da redução sob carga axial. Fraturas em cunha são ideais para redução indireta, em que a placa submoldada, posicionada no plano de cisalhamento, realiza a redução da fratura ao colocarmos como primeiro parafuso o do orifício junto ao vértice.

**Fraturas laterais:** Nas fraturas unicondilares laterais simples, utiliza-se acesso lateral direto com extensão anterolateral até a tuberosidade anterior da tíbia. Dependendo da localização do traço, o acesso anterolateral pode oferecer visualização superior, sobretudo quando há impacção. A placa pode ser passada de forma submuscular e os parafusos proximais são inseridos percutaneamente.

**Fraturas mediais:** Nas fraturas unicondilares mediais, o acesso subvasto medial proporciona excelente exposição, incluindo fraturas coronais (Hoffa). A redução geralmente ocorre sob tração, com joelho em extensão e estresse valgo (ou varo para fraturas laterais).

Na presença de impacções e quando a fratura tem um plano mais oblíquo, vias de acesso alternativas, posteromediais ou posterolaterais podem ser necessárias. 11

### Fraturas bicondilares – articulares totais (AO 33C)

As fraturas articulares totais mais frequentes são as AO C2 e C3, que apresentam cominuição metafisária. O DCS trouxe benefício de estabilidade angular metafisária, permanecendo ainda útil atualmente. Existe elevada incidência de traços coronais tipo Hoffa (em torno de 38%), 9 11 21 tornando o uso de placas anatômicas bloqueadas mais recomendado nesses casos devido à variedade de orifícios para parafusos distais nos côndilos, evitando conflitos com os parafusos inseridos em outras direções na fixação do componente de Hoffa.

**Fig. 1:**
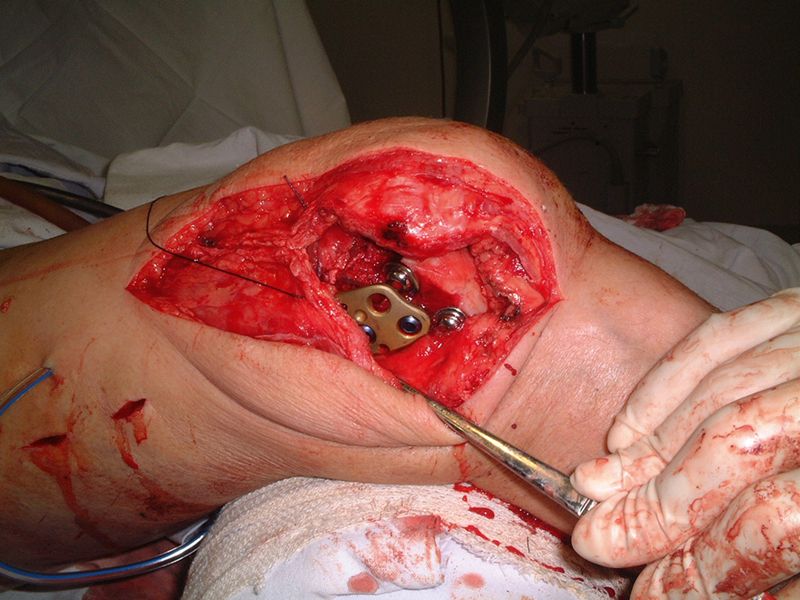
Imagem intraoperatória mostrando os parafusos de tração, com 6,5 mm, colocados estrategicamente na periferia do côndilo lateral do fêmur, evitando a posição em que foi colocada, na sequência, a placa lateral. Acesso *transarticular approach and retrograde plate osteosynthesis* (TARPO), anterior, e parapatelar lateral. A tendência atual é utilizar parafusos de tração 3,5 mm, que ocupam menor espaço, com a técnica de compressão que utiliza o canal de deslizamento.

A via de acesso anterolateral ( *transarticular approach and retrograde plate osteosynthesis* , TARPO, em inglês) 22 permite ampla exposição articular e fixação com placa pelo mesmo acesso na maioria dos casos. A via anteromedial pode ser preferida quando a cominuição maior é medial, podendo-se associar abordagens combinadas, com dupla fixação medial e lateral. Com os acessos anteriores, a osteotomia da tuberosidade anterior da tíbia, que era feita em casos complexos na via de acesso lateral, tornou-se desnecessária. A articulação é reduzida com fios de Kirschner e parafusos de pequenos fragmentos são colocados perifericamente para permitir o posicionamento adequado da placa lateral. Parafusos de 3,5 mm são preferidos por ocuparem menos espaço que os parafusos para osso esponjoso de 6,5 mm. É importante garantir que parafusos longos 3,5 mm estejam disponíveis, pois alguns instrumentais disponibilizam parafusos de comprimento máximo de 50 mm. Evita-se, sempre que possível, atravessar a cartilagem; quando necessário, escareia-se o ponto de entrada para sepultar a cabeça dos parafusos, ou utilizam-se parafusos pequenos sem cabeça. Fragmentos menores osteocondrais podem requerer implantes de minifragmentos e placas horizontais. 23 24 Após a fixação articular, procede-se à redução metafisária indireta sob intensificador, preservando-se ao máximo a vascularização ( Fig. 1 ). O alinhamento frontal e sagital deve ser checado repetidamente, utilizando o eixo mecânico por fluoroscopia. 19 O desvio mais comum é valgo associado ao recurvato.

Atualmente, os implantes mais empregados são hastes retrógradas com múltiplos bloqueios distais e placas laterais bloqueadas. 4 7 9 25 26 27 Hastes retrógradas são apropriadas para fraturas de 33C1 a 33C2, porém são mais arriscadas nas fraturas 33C3, por causa da fragmentação articular. O controle de alinhamento com hastes é mais difícil e pode requerer parafusos *poller* . Pequenas placas para redução metafisária também podem complementar a fixação e serem utilizadas conjuntamente com as hastes. Em idosos, preferem-se implantes longos, protegendo todo o osso.

Metanálises recentes apresentam resultados semelhantes entre hastes e placas quanto à consolidação, complicações, reoperações e tempo cirúrgico, com pequenas diferenças favoráveis às placas em mobilidade e qualidade de redução, porém com mais distúrbios de consolidação. 8 28 A escolha deve considerar a complexidade dos traços articulares, dificuldade no controle da redução (superioridade das placas), necessidade de apoio precoce (vantagem para hastes) e condições clínicas.

### Duplo implante

**Fig. 2:**
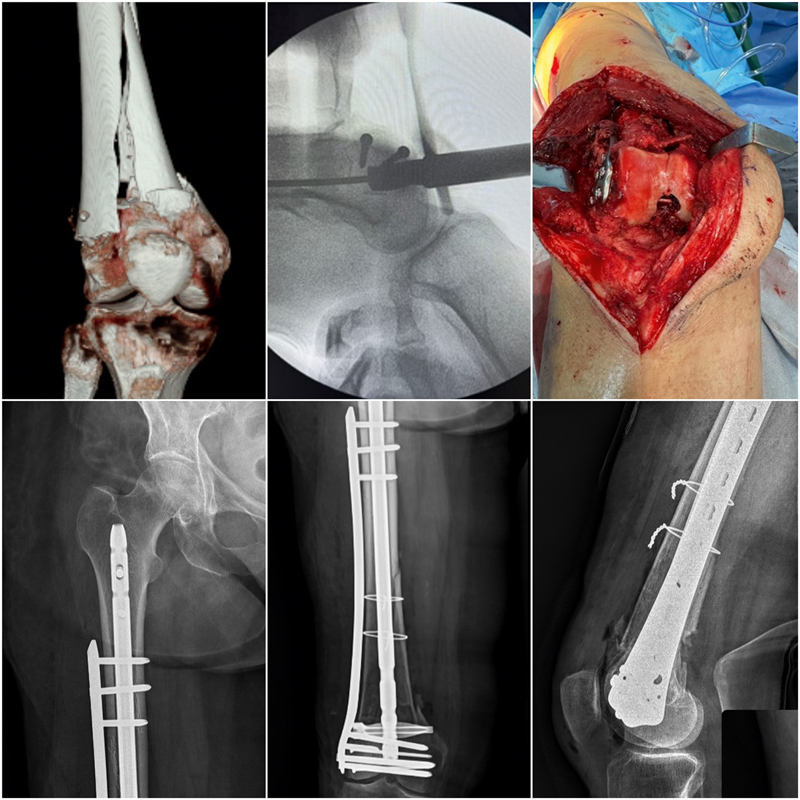
Imagens intraoperatórias de uma fratura distal do fêmur em osso osteoporótico de uma paciente de 69 anos, transferida 9 dias após o trauma inicial, com a fratura fixada provisoriamente. Observa-se a estratégia de redução pelo acesso anterior TARPO, fixação da fratura articular com parafusos fora do trajeto da haste intramedular longa, que ultrapassa a altura do trocanter menor. Por fim, a placa lateral é ajustada com os parafusos suplementando a fixação articular e os parafusos divergindo da região proximal da haste. A utilização de 2 fios de cerclagem em aço na diáfise facilitou a redução da fratura e aumentou a estabilidade da fixação.

A osteossíntese com duplo implante (placa lateral associada a placa medial, ou placa medial ou lateral combinada com haste retrógrada) tem ganhado destaque nas situações de falta de contato ou suporte medial, ossos osteoporóticos, pacientes obesos, pacientes pouco colaborativos (alcoolismo, demência, abuso de drogas), ou fraturas periprotéticas. 4 5 6 26 29 30 31 A dupla fixação com placas tem a vantagem de um controle melhor da qualidade da redução, mas às custas de maior disseção, perda sanguínea levemente maior e mais distúrbios de consolidação. A combinação haste-placa oferece melhor estabilidade mecânica, favorecendo descarga precoce, e está associada à maiores taxas de consolidação, sendo útil inclusive em pseudoartroses. 5 6 A utilização de hastes combinadas com placas carrega as vantagens de ambos os métodos: ao mesmo tempo em que a resistência torcional e a qualidade da redução de traços articulares são melhores com placas, as hastes proporcionam vantagens biomecânicas pela sua posição central e maior estabilidade axial, tornando mais segura a descarga precoce completa de peso, o que se mostra muito benéfico nos pacientes idosos. Obviamente, o emprego de ambos os implantes demanda treinamento e cuidados durante a colocação dos parafusos, para que não ocorra conflito com a haste, que normalmente é inserida antes da colocação da placa. A ordem normalmente é primeiro reduzir e fixar os fragmentos articulares, depois posicionar a haste e, por fim, adicionar a placa ( Fig. 2 ).

### Artroplastia do joelho como opção à fixação

Em pacientes idosos, com osso osteoporótico e fraturas altamente cominutivas, ou em algumas fraturas periprotéticas com soltura do componente femoral, as taxas de complicações após osteossíntese (falhas de fixação, pseudoartroses e reoperações) são relevantes. Nessas situações, quando o sucesso da fixação for considerado improvável, a artroplastia do joelho, seja na forma de artroplastia total primária ou de substituição distal do fêmur, 32 33 surge como alternativa para permitir mobilização precoce, descarga de peso e redução do tempo de imobilização, fatores cruciais em pacientes com múltiplas comorbidades. Geralmente os implantes protéticos utilizados têm maior constricção, para compensar a instabilidade ligamentar resultante das fraturas. Quando há soltura do componente femoral, a substituição completa da região distal do fêmur pode ser opção. Todavia, há que ser considerado que implantes especiais têm restrições quanto à disponibilidadee são cirurgias tecnicamente mais exigentes e de maior custo. Idealmente, esses casos devem ser concentrados em centros com experiência em artroplastia complexa e com acesso a próteses modulares e aumentos metafisários. Obviamente, as complicações e a mortalidade são mais altas que nos pacientes submetidos a artroplastias primárias do joelho por osteoartrite. Revisões recentes mostram que as artroplastias estão mais associadas com complicações clínicas do que a fixação interna. 34 35

## Situações especiais

### Fraturas expostas

Fraturas expostas representam de 11 a 14% das fraturas distais do fêmur de baixa energia e até 55% das de alta energia, apresentando maior risco de infecção, falhas de fixação e distúrbios de consolidação. 36 Exigem atendimento imediato, seguindo todos os princípios de tratamento de fraturas expostas, com antibioticoprofilaxia precoce, desbridamento no centro cirúrgico e rápida cobertura da articulação por partes moles para prevenir infecção. A síntese articular mínima pode facilitar reconstruções posteriores, desde que não comprometa a fixação definitiva. Os parafusos devem evitar trajetos futuros de placas ou hastes retrógradas e novas vias de acesso devem ser evitadas, para não agravar a desvascularização.

**Fig. 3:**
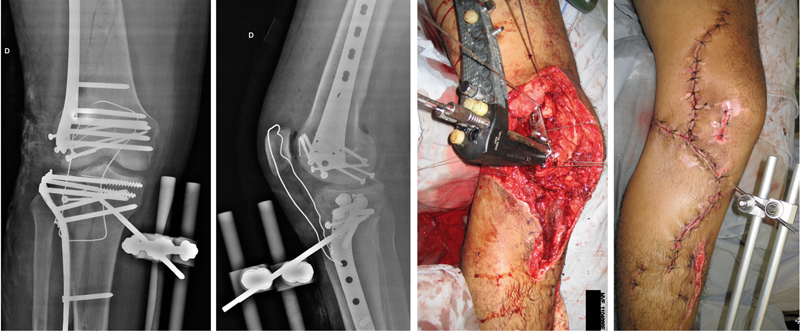
Osteossíntese de fratura bicondilar 33C3 exposta, mostrando a colocação periférica de parafusos de pequenos fragmentos 3,5 mm (também fixando o componente de Hoffa lateral) e placa com parafusos bloqueados. Apesar do trauma de alta energia e lesão de partes moles, ainda foi possível o fechamento primário da pele.

A fixação inicial deve ser rápida, com parafusos de pequenos fragmentos, pela mesma via do desbridamento, realizada idealmente pelo cirurgião responsável pela futura etapa definitiva. Outras estratégias incluem antibioticoterapia local com pérolas de polimetilmetacrilato, curativos a vácuo e uso de espaçadores de cimento quando for planejada a técnica de Masquelet para falhas metafisárias. 37 O prognóstico das fraturas expostas é pior, com maiores taxas de infecção, distúrbios de consolidação e mais reoperações ( Fig. 3 ).

### Fraturas de Hoffa

As fraturas de Busch-Hoffa são frequentes em fraturas bicondilares complexas, 38 embora possam ocorrer isoladamente. Ocorrem em mais de 30% das fraturas do tipo C e podem passar despercebidas em até 25% dos casos. 11 O traço no plano coronal exige acessos específicos quando os fragmentos são mais posteriores ou quando existem fragmentos osteocondrais pequenos. Além do típico fragmento em cunha por cisalhamento, podem existir áreas de depressão condral com fragmentos interpostos. Quando fazem parte de fraturas complexas, os traços de Hoffa são geralmente fixados com parafusos de tração de anterior para posterior, com redução facilitada pela extensão do fragmento distal durante a redução do maciço condilar através da própria via de acesso anterior. Em fragmentos pequenos posteriores, parafusos de posterior para anterior oferecem maior estabilidade. Fragmentos com extensão metafisária devem ser apoiados por placas de suporte para resistir a cargas axiais, utilizando acessos posterolaterais ou posteromediais. Placas de pequenos fragmentos posicionadas horizontalmente também aumentam a estabilidade. 11 24

**Fig. 4:**
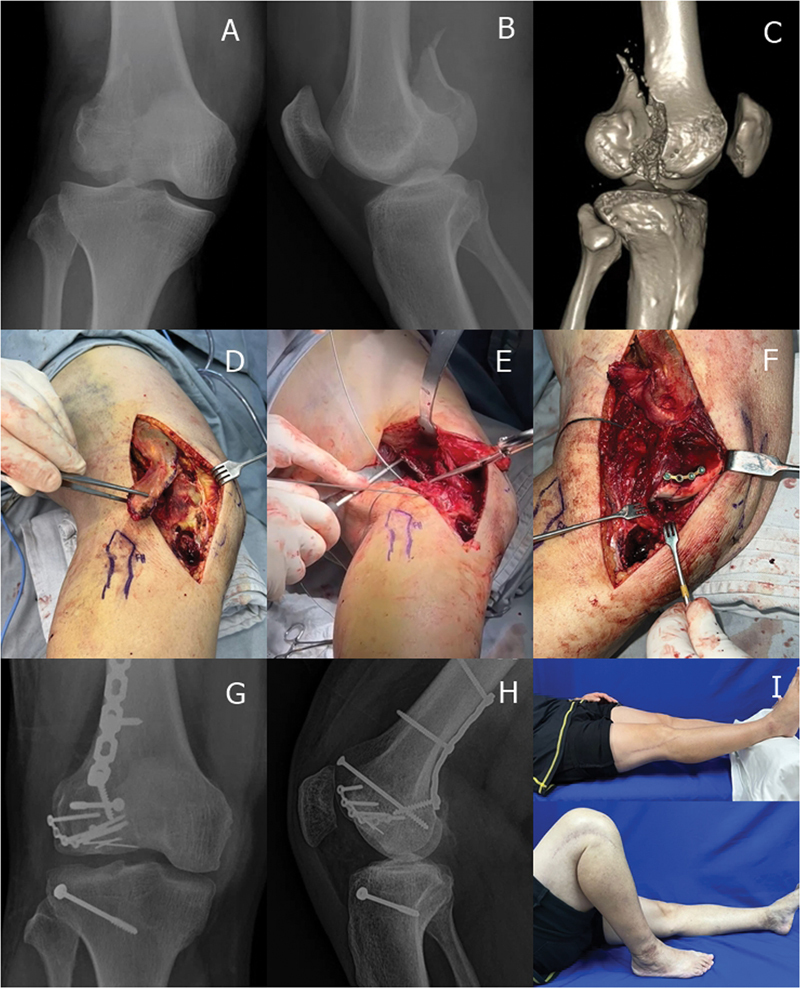
Fratura coronal do côndilo lateral do fêmur (fratura de Hoffa) ( **A** , **B** ); variante do tipo I de Letenneur ( **C** – fragmento intercalado). Neste caso, foi realizada uma via posterolateral com o paciente em decúbito lateral, onde a osteotomia do tubérculo de Gerdy ( **D** ) permitiu uma visualização direta da fragmentação articular, assim como da região metafisária para colocação da placa de suporte ( **E** ). Note a utilização de uma pequena placa de minifragmentos posicionada horizontalmente (4F), para melhorar a estabilidade da superfície articular. Radiografias pós-operatórias ( **G** , **H** ); mobilidade articular aos 5 meses de pós-operatório ( **I** ).

A Classificação de Letenneur 11 auxilia o planejamento cirúrgico:

Tipo I: traço alinhado à cortical posterior; fragmentos grandes. A variante envolve cominuição ou depressão articular. A fixação mais estável envolve placa de suporte 3,5 mm no vértice da fratura e parafusos de posterior para anterior, preferencialmente por via posterolateral, com proteção do nervo fibular. Nas variantes com fragmentos deprimidos, o acesso lateral associado à osteotomia do tubérculo de Gerdy permite visualização e redução destes fragmentos ( Fig. 4 ).

Tipo II: fragmentos menores e mais posteriores, subdivididos em IIa, IIb e IIc conforme o tamanho. Por serem mais posteriores e com fragmentos pequenos, exigem acessos posteriores, com inserção dos parafusos muitas vezes através da cartilagem.

Tipo III: traço articular mais oblíquo e anterior. São geralmente acessadas por via anterolateral, com fixação de anterior para posterior.

Fraturas isoladas do côndilo medial são menos comuns, sendo o acesso subvasto medial o mais indicado; raramente é necessário um acesso posterior entre o gastrocnêmio medial e o grácil.

### Fraturas periprotéticas

As fraturas periprotéticas ocorrem em 0,3 a 5,5% nas artroplastias primárias e atingem até 30% nas revisões. 29 39 A maioria ocorre após traumas de baixa energia, sendo mais comuns em pacientes idosos e do sexo feminino. Outros fatores de risco incluem uso de corticoides, comorbidades, mau alinhamento e erros intraoperatórios. Recentemente, fraturas associadas a orifícios de pinos de navegação robótica também foram relatadas.

Nas fraturas periprotéticas do joelho, a osteoporose e o baixo estoque ósseo dificultam a fixação, especialmente quando o componente femoral da prótese tem sua região intercondilar fechada, característica encontrada em algumas próteses com estabilização posterior (tipo *posterior stabilized* ). A avaliação da estabilidade do componente é essencial, sendo a tomografia com supressão metálica uma ferramenta importante. Havendo soltura, a revisão protética é obrigatória; no Reino Unido, cerca de 3,8% das revisões decorrem dessas fraturas.

O Sistema Unificado de Classificação (Unified Classification Sytstem, UCS, em inglês) tem ganhado adoção crescente, mas a classificação Lewis-Rorabeck segue ainda como a mais utilizada: 30

- Tipo I: fratura sem desvio, prótese fixa;
- Tipo II: fratura desviada, prótese fixa; e
- Tipo III: soltura do componente femoral.

**Fig. 5:**
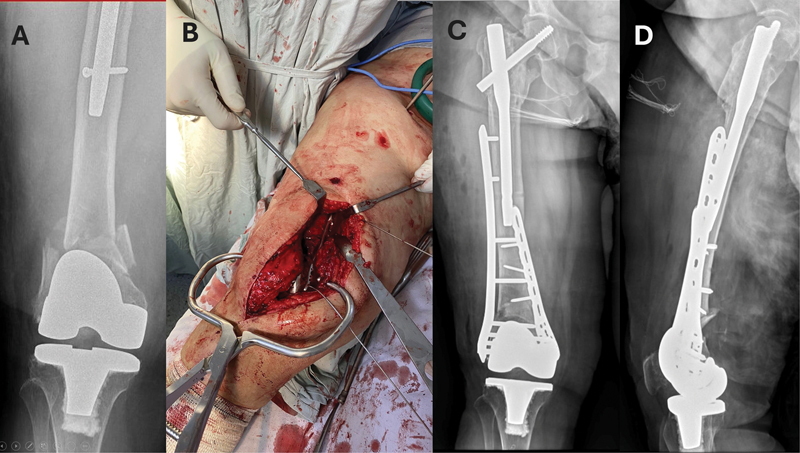
Fratura interprotética da região distal do fêmur ( **A** ), fixada com 2 placas bloqueadas. A fotografia intraoperatória ( **B** ) ilustra o acesso medial e a redução com o clampe periarticular grande. Foi realizada uma via anteromedial mais ampla e um segundo acesso lateral para inserção percutânea da placa lateral. As figuras seguintes mostram as radiografias em incidência anteroposterior (AP) ( **C** ), lateral ( **D** ). É importante evitar que as placas terminem na mesma região ou criem uma zona de concentração de estresses entre a haste e as placas. Foram utilizados parafusos especiais periprotéticos na região proximal da placa lateral.

Nos tipos I e II, observa-se tendência ao uso de duas placas ou de placas combinadas com hastes intramedulares, empregando implantes longos que protejam toda a extensão femoral. A placa lateral é inserida por via lateral direta, submuscular, com parafusos proximais percutâneos; a placa medial é geralmente menor e introduzida por acesso subvasto medial ( Fig. 5 ). Quando a placa medial é longa, há necessidade de se evitar risco à artéria femoral mediante torção proximal da placa. Em casos selecionados, uma haste retrógrada longa pode ser utilizada isoladamente ou combinada com placas laterais. É fundamental confirmar previamente se o componente femoral da prótese permite a passagem da haste retrógrada (caixa aberta). Um problema relativamente comum é o deslocamento posterior do ponto de entrada da haste devido ao desenho do componente femoral, o que pode resultar em deformidade em recurvato. Para solucionar essa limitação, foram desenvolvidas hastes intramedulares retrógradas específicas para fraturas periprotéticas, com maior angulação distal, permitindo um ponto de entrada mais posterior sem induzir ao recurvato.

Nas fraturas tipo III, com soltura protética, podem ser usados componentes protéticos femorais de haste longa, preferencialmente associados à uma placa lateral. Na presença de cominuição grave ou perda óssea extensa, pode-se recorrer a próteses de ressecção (substituição de toda a região distal do fêmur) ou revisão com aloenxertos estruturais, quando disponíveis.

## Reabilitação

A mobilização articular precoce deve ser incentivada e recursos como a mobilização passiva contínua (MPC) podem auxiliar no ganho progressivo de arco de movimento. Uma analgesia eficaz é fundamental para a recuperação funcional. A profilaxia antitrombótica é mantida por 10 a 30 dias. A deambulação precoce com duas muletas ou andador deve ser estimulada. Evitar apoio por tempo prolongado é prejudicial, podendo causar distrofia, complicações vasculares e atrasos na consolidação. É seguro autorizar descarga de peso conforme tolerado em fraturas periprotéticas tratadas com placas ou hastes, 40 mesmo em idosos. A utilização de duplo implante aumenta ainda mais a estabilidade sob carga axial.

## Resultados e complicações

Os resultados funcionais das fraturas distais do fêmur variam conforme o padrão da fratura, presença de lesões associadas, técnica cirúrgica e qualidade da reabilitação. Quando obtida redução anatômica e estabilidade adequada, a maioria dos pacientes recupera mobilidade articular suficiente para realizar atividades da vida diária. Entretanto, fraturas articulares complexas frequentemente evoluem com algum grau de rigidez e dor residual. As complicações mais temidas são as infecções pós-operatórias, os distúrbios de consolidação e as falhas de fixação. No longo prazo, o risco de osteoartrite pós-traumática sempre deve ser considerado.

## Considerações finais

As fraturas distais do fêmur continuam desafiadoras, e este capítulo buscou sintetizar os principais aspectos envolvidos no seu tratamento. Em sua distribuição epidemiológica bimodal, temos os pacientes jovens, vítimas de traumas de alta energia, e indivíduos idosos, em geral portadores de múltiplas comorbidades e com recuperação funcional mais lenta e complexa. Apesar dos progressos obtidos no desenvolvimento de implantes intramedulares modernos e placas anatômicas com parafusos bloqueados, a morbidade e a mortalidade associadas a essas fraturas ainda permanecem relevantes.

Foram discutidas situações complexas, incluindo fraturas expostas, fraturas de Hoffa e as fraturas periprotéticas, todas elas demandando estratégias diferentes. Entre as tendências atuais, merece destaque o uso de dupla fixação, que se mostra particularmente vantajosa nos padrões mais cominutivos, na falha de contato ósseo medial, nos ossos osteoporóticos e nos casos de fraturas periprotéticas.

Por fim, o sucesso do tratamento não depende apenas da estabilização óssea, mas também da reabilitação adequada, o que inclui descarga de peso precoce e mobilização articular, especialmente na população idosa.

## References

[1] Singh R Ambade R Landge S Goyal S Goel S Comprehensive Review on Distal Femur Fractures: From Epidemiology to Treatment Strategies Cureus 2024 16 04 e57937 10.7759/cureus.57937 38738010 PMC11084923

[2] Von Rehlingen-Prinz F Eggeling L Dehoust J Current standard of care for distal femur fractures in Germany and Switzerland Injury 2023 54 10 110936 10.1016/j.injury.2023.110936 37516571

[3] Tsai S HL Lin T Y Tischler E H Distal femur fractures have a higher mortality rate compared to hip fractures among the elderly: Insights from the National Trauma Data Bank Injury 2021 52 07 1903 1907 10.1016/j.injury.2021.04.023 33896612

[4] Wright D J DeSanto D J McGarry M H Lee T Q Scolaro J A Supplemental Fixation of Supracondylar Distal Femur Fractures: A Biomechanical Comparison of Dual-Plate and Plate-Nail Constructs J Orthop Trauma 2020 34 08 434 440 10.1097/bot.0000000000001749 32032183

[5] Mueller G EM Nail-Plate Constructs for Periprosthetic Distal Femur Fractures J Knee Surg 2019 32 05 403 406 10.1055/s-0039-1683444 30919389

[6] Kontakis M G Giannoudis P V Nail plate combination in fractures of the distal femur in the elderly: A new paradigm for optimum fixation and early mobilization? Injury 2023 54 02 288 291 10.1016/j.injury.2022.11.035 36400628

[7] Tripathy S K Mishra N P Varghese P Dual-Plating in Distal Femur Fracture: A Systematic Review and Limited Meta-analysis Indian J Orthop 2021 56 02 183 207 10.1007/s43465-021-00489-0 35140850 PMC8789962

[8] Neradi D Sodavarapu P Jindal K Kumar D Kumar V Goni V Locked Plating Versus Retrograde Intramedullary Nailing for Distal Femur Fractures: a Systematic Review and Meta-Analysis Arch Bone Jt Surg 2022 10 02 141 152 10.22038/abjs.2021.53515.2656 35655740 PMC9117898

[9] Beltran M J Gary J L Collinge C A Management of distal femur fractures with modern plates and nails: state of the art J Orthop Trauma 2015 29 04 165 172 10.1097/bot.0000000000000302 25793566

[10] Tosun B Medial approach for the treatment of femur fractures in association with vascular injury Injury 2020 51 06 1367 1372 10.1016/j.injury.2020.03.044 32336478

[11] Pires R E Giordano V Fogagnolo F Yoon R S Liporace F A Kfuri M Algorithmic treatment of Busch-Hoffa distal femur fractures: A technical note based on a modified Letenneur classification Injury 2018 49 08 1623 1629 10.1016/j.injury.2018.06.008 29885965

[12] Brodke D J Devana S K Upfill-Brown A Lee C Cost-effectiveness of fixation versus arthroplasty for geriatric distal femur fractures Injury 2022 53 02 661 668 10.1016/j.injury.2021.11.054 34887075 PMC10400013

[13] Chuluunbaatar Y Benachar N Khroud-Dhillon H Srinivasan A Rojoa D Raheman F Early and 1-year mortality of native geriatric distal femur fractures: A systematic review and time-to-event meta-analysis J Clin Orthop Trauma 2024 50 102375 10.1016/j.jcot.2024.102375 38495682 PMC10943051

[14] Moloney G B Pan T Van Eck C F Patel D Tarkin I Geriatric distal femur fracture: Are we underestimating the rate of local and systemic complications? Injury 2016 47 08 1732 1736 10.1016/j.injury.2016.05.024 27311551

[15] Myers P Laboe P Johnson K J Patient Mortality in Geriatric Distal Femur Fractures J Orthop Trauma 2018 32 03 111 115 10.1097/bot.0000000000001078 29462121

[16] Court-Brown C M Caesar B Epidemiology of adult fractures: A review Injury 2006 37 08 691 697 10.1016/j.injury.2006.04.130 16814787

[17] Griffin X L Parsons N Zbaeda M M McArthur J Interventions for treating fractures of the distal femur in adults Cochrane Database Syst Rev 2015 2015 08 CD010606 10.1002/14651858.CD010606.pub2 26270891 PMC9207810

[18] Merino-Rueda L R Rubio-Sáez I Mills S Rubio-Suárez J C Mortality after distal femur fractures in the elderly Injury 2021 52 04 S71 S75 10.1016/j.injury.2021.03.066 33992422

[19] Scott B R Wright R D Moghadamian E S Intraoperative Assessment of Coronal Alignment in Distal Femur Fracture Fixation: Technical Trick J Orthop Trauma 2019 33 02 e69 e72 10.1097/BOT.0000000000001334PubMed 30277984

[20] Paccola C A Supracondylar fracture of the femur. A method of avoiding the malalignment of the distal femur during internal fixation Unfallchirurg 1989 92 08 379 380 2799395

[21] Ehlinger M Ducrot G Adam P Bonnomet F Distal femur fractures. Surgical techniques and a review of the literature Orthop Traumatol Surg Res 2013 99 03 353 360 10.1016/j.otsr.2012.10.014 23518071

[22] Krettek C Miclau T Stephan C Tscheme H Transarticular Approach and Retrograde Plate Osteosynthesis (TARPO): An Alternative Surgical Approach for Complex Distal Intra-Articular Femur Fractures Tech Orthop 1999 14 03 219 229 10.1097/00013611-199909000-00008

[23] Pires R E Rabelo J MG Cimini C A Biomechanics of internal fixation in Hoffa fractures - A comparison of four different constructs Injury 2024 55 02 111219 10.1016/j.injury.2023.111219 38029682

[24] Pires R E Giordano V Santos JKd Labronici P J Andrade MAd Lourenço PRdT Expanding indications of the horizontal belt plate: a technical note Injury 2015 46 10 2059 2063 10.1016/j.injury.2015.06.024 26115580

[25] Piétu G Ehlinger M Minimally invasive internal fixation of distal femur fractures Orthop Traumatol Surg Res 2017 103(1S):S161 S169 10.1016/j.otsr.2016.06.025 27867137

[26] Canton G Giraldi G Dussi M Ratti C Murena L Osteoporotic distal femur fractures in the elderly: peculiarities and treatment strategies Acta Biomed 2019 90(12-S):25 32 10.23750/abm.v90i12-S.8958 PMC7233703 31821280

[27] Von Keudell A Shoji K Nasr M Lucas R Dolan R Weaver M J Treatment Options for Distal Femur Fractures J Orthop Trauma 2016 30 02 S25 S27 10.1097/BOT.0000000000000621 27441931

[28] Aggarwal S Rajnish R K Kumar P Srivastava A Rathor K Haq R U Comparison of outcomes of retrograde intramedullary nailing versus locking plate fixation in distal femur fractures: A Systematic Review and Meta-analysis of 936 patients in 16 studies J Orthop 2022 36 36 48 10.1016/j.jor.2022.12.007 36591439 PMC9800249

[29] Medda S Kessler R B Halvorson J J Pilson H T Babcock S Carroll E A Technical Trick: Dual Plate Fixation of Periprosthetic Distal Femur Fractures J Orthop Trauma 2021 35 04 e148 e152 10.1097/bot.0000000000001869 32569069

[30] Al-Jabri T Ridha M McCulloch R A Jayadev C Kayani B Giannoudis P V Periprosthetic distal femur fractures around total knee replacements: A comprehensive review Injury 2023 54 04 1030 1038 10.1016/j.injury.2023.02.037 36854630

[31] Beeres F JP Emmink B L Lanter K Link B C Babst R Minimally invasive double-plating osteosynthesis of the distal femur Oper Orthop Traumatol 2020 32 06 545 558 10.1007/s00064-020-00664-w 32548732

[32] Salazar B P Babian A R DeBaun M R Distal Femur Replacement Versus Surgical Fixation for the Treatment of Geriatric Distal Femur Fractures: A Systematic Review J Orthop Trauma 2021 35 01 2 9 10.1097/bot.0000000000001867 32569072

[33] Lizcano J D Giakas A M Goh G S Abbaszadeh A Reddy Y C Courtney P M Fix or Replace? Comparable Outcomes With Internal Fixation and Distal Femoral Replacement for Periprosthetic Fractures Above Total Knee Arthroplasty J Arthroplasty 2025 40 04 1048 10540 10.1016/j.arth.2024.10.006 39428002

[34] Bundschuh K E Grommersch B M Tipton S C Chihab S Wilson J M Guild G N III Distal Femoral Replacement versus Operative Fixation for Periprosthetic Distal Femur Fractures: A Systematic Review and Meta-Analysis J Arthroplasty 2023 38(7, Suppl 2)S450 S458 10.1016/j.arth.2023.01.044 36738864

[35] Wadhwa H Salazar B P Goodnough L H Distal Femur Replacement Versus Open Reduction and Internal Fixation for Treatment of Periprosthetic Distal Femur Fractures: A Systematic Review and Meta-Analysis J Orthop Trauma 2022 36 01 1 6 10.1097/BOT.0000000000002141 34001801

[36] Moloney G Tarkin I S Optimal Management of the Patient With an Open Distal Femur Fracture Oper Tech Orthop 2018 28 03 118 124 10.1053/j.oto.2018.07.002

[37] Kalantar S H Saffar H Hoveidaei A H Bone reconstruction with modified Masquelet technique in open distal femoral fractures: a case series BMC Musculoskelet Disord 2024 25 01 26 10.1186/s12891-023-07091-5 38167118 PMC10759597

[38] Nork S E Segina D N Aflatoon K The association between supracondylar-intercondylar distal femoral fractures and coronal plane fractures J Bone Joint Surg Am 2005 87 03 564 569 10.2106/JBJS.D.01751 15741623

[39] Wallace S S Bechtold D Sassoon A Periprosthetic fractures of the distal femur after total knee arthroplasty : Plate versus nail fixation Orthop Traumatol Surg Res 2017 103 02 257 262 10.1016/j.otsr.2016.11.018 28089667

[40] Striano B M Grisdela P T Jr Shapira S Heng M Early Weight Bearing after Distal Femur Fracture Fixation Geriatr Orthop Surg Rehabil 2022 13:21514593211070128 10.1177/21514593211070128 PMC8801638 35111355

